# Supplementary material for: Can Microfluidics Improve Sperm Quality? A Prospective Functional Study
Source: Biomedicines. 2024 May 20;12(5):1131. doi: 10.3390/biomedicines12051131 (PMC11118748; doi:10.3390/biomedicines12051131)
Supplement: Supplementary file 1 [file biomedicines-12-01131-s001.zip › biomedicines-2993196-supplementary.pdf]

biomedicines

MDPI

Supplementary Materials:

Table S1. How sperm quality parameters influence DNA framentation.

Variables	Total	<5.4	5.4–8.86	8.87–13.76	≥13.77	*p*-value
**Sample size**	181 (100%)	45 (24.86 %)	44 (24.31 %)	46 (25.41 %)	46 (25.41 %)	
**Sperm Concentration Median (IQR)**	43.00 (29.10–65.90)	47.00 (34.00–72.00)	41.55 (30.30–64.38)	45.15 (31.40–79.38)	39.50 (24.60–51.62)	0.256
**Progressive Motile Sperm Cells (%) Median (IQR)**	43.00 (35.00–51.00)	48.00 (37.00–55.00)	44.50 (39.00–54.25)	41.50 (34.25–47.00)	36.00 (32.25–46.50)	0.002
**Total Progressive Motile Sperm Count Median (IQR)**	58.30 (30.78–89.90)	59.40 (35.28–112.50)	62.31 (36.25–90.48)	66.35 (33.48–108.02)	42.97 (27.51–72.61)	0.075
**Sperm Vitality (%) Median (IQR)**	81.00 (75.00–85.00)	82.00 (79.00–87.00)	83.00 (79.00–88.00)	77.00 (71.00–83.00)	78.00 (71.00–82.75)	<0.001
**Normal Morphology (%) Median (IQR)**	2.00 (1.00–3.00)	2.00 (1.00–3.00)	2.00 (1.00–2.25)	2.00 (2.00–3.00)	2.00 (1.00–3.00)	0.143
**Sperm Chromatin Stability (%) Median (IQR)**	75.00 (67.00–82.00)	79.00 (73.00–84.00)	79.00 (68.00–88.00)	72.50 (67.50–77.25)	68.50 (61.75–78.00)	<0.001

* Semen quality parameters by quartile of sperm DNA fragmentation index in unprocessed samples.

Table S2. SwimCount™ Harvester vs Density Gradient Centrifugation by quartiles.

Sperm Quality Parameters Sample Size	Quartiles	Unprocessed	SwimCount™ Harvester	DGC	*p*-value a	*p*-value b	*p*-value c
Sperm Concentration (mill/mL) Median (IQR) *n* =100	Q1	12.70 (5.65–18.35)	1.80 (0.85–2.95	1.60 (0.45–2.85)	$p < 0.001$	$p < 0.001$	$p = 1.00$
	Q2	35.20 (28.00–37.15)	5.40 (4.65–6.80)	5.00 (3.75–6.20)	$p < 0.001$	$p < 0.001$	$p = 0.652$
	Q3	47.00 (41.80–52.30)	10.20 (8.70–13.25)	9.80 (7.95–12.65)	$p < 0.001$	$p < 0.001$	$p = 1.00$
	Q4	95.00 (76.70–117.5)	24.00 (19.05–32.70)	18.60 (17.60–26.50)	$p < 0.001$	$p < 0.001$	$p = 0.728$
Progressive Motile Sperm Cells (%) Median (IQR) *n* = 100	Q1	29.00 (24.00–31.00)	70.00 (64.50–74.00)	65.00 (52.00–67.00)	$p < 0.001$	$p < 0.001$	$p = 0.083$
	Q2	38.00 (36.50–40.00)	78.00 (76.00–79.00)	72.00 (70.50–73.50)	$p < 0.001$	$p < 0.001$	$p < 0.001$
	Q3	45.00 (43.00–46.00)	81.00 (80.50–83.00)	77.00 (76.00–78.00)	$p < 0.001$	$p < 0.001$	$p < 0.001$
	Q4	55.00 (51.50–57.50)	86.00 (85.50–87.50)	81.00 (80.00–86.00)	$p < 0.001$	$p < 0.001$	$p = 0.021$
Total Progressive Motile Sperm Count (mill) Median (IQR) *n* = 100	Q1	3.73 (2.25–18.15)	1.14 (0.59–1.78)	0.74 (0.22–1.10)	$p < 0.001$	$p < 0.001$	$p = 0.180$
	Q2	36.90 (30.36–43.10)	3.26 (2.84–3.77)	2.07 (1.63–2.32)	$p < 0.001$	$p < 0.001$	$p < 0.001$
	Q3	65.44 (58.81–73.24)	6.26 (5.38–8.48)	3.94 (3.08–4.74)	$p < 0.001$	$p < 0.001$	$p = 0.001$
	Q4	135.8 (106.5–189.6)	14.72 (11.92–20.72)	9.24 (6.89–11.43)	$p < 0.001$	$p < 0.001$	$p = 0.009$
DNA Fragmentation Index (%) Median (IQR) DGC *n* = 90	Q1	4.74 (3.71–5.92)	1.28 (0.86–1.54)	4.47 (3.12–5.63)	$p < 0.001$	$p = 1.00$	$p < 0.001$
	Q2	9.42 (8.65–9.88)	2.85 (2.30–3.38)	9.90 (7.75–10.98)	$p < 0.001$	$p = 1.00$	$p < 0.001$
	Q3	12.05 (10.97–12.97)	5.76 (4.45–6.26)	16.24 (13.91–19.31)	$p < 0.001$	$p = 0.003$	$p < 0.001$
	Q4	19.58 (16.21–23.39)	12.67 (11.15–20.32)	30.93 (25.25–41.86)	$p = 0.032$	$p = 0.002$	$p < 0.001$

* Quartile stratification of sperm quality parameters to compare SwimCount™ Harvester with Density Gradient Centrifugation. a Statistically significant differences between unprocessed samples and samples processed by SwimCount™ Harvester. b Statistically significant differences between unprocessed samples and samples processed by DGC. c Statistically significant difference between SwimCount™ Harvester and DGC.

Table S3. SwimCount™ Harvester vs Swim-up by quartiles.

Sperm Quality Parameters Sample Size	Quartiles	Unprocessed	SwimCount™ Harvester	Swim-up	*p*-value a	*p*-value b	*p*-value c
**Sperm Concentration (mill/mL) Median (IQR) *n* =100**	Q1	24.00 (19.95–25.60)	6.80 (4.05–7.95)	3.00 (2.40–4.00)	$p < 0.001$	$p < 0.001$	$p = 0.012$
	Q2	38.00 (32.90–43.50)	10.60 (9.75–12.85)	7.00 (5.85–8.20)	$p < 0.001$	$p < 0.001$	$p < 0.001$
	Q3	55.00 (50.40–59.50)	19.20 (16.50–21.05)	11.80 (9.65–12.75)	$p < 0.001$	$p < 0.001$	$p < 0.001$
	Q4	88.10 (73.50–92.00)	30.70 (28.60–36.35)	22.40 (16.65–26.80)	$p < 0.001$	$p < 0.001$	$p = 0.004$
**Progressive Motile Sperm Cells (%) Median (IQR) *n* = 100**	Q1	31.00 (24.50–34.50)	82.00 (80.00–84.00)	80.00 (76.00–82.00)	$p < 0.001$	$p < 0.001$	$p = 0.320$
	Q2	41.00 (36.00–42.00)	87.00 (86.00–87.50)	86.00 (85.00–87.00)	$p < 0.001$	$p < 0.001$	$p = 0.046$
	Q3	50.00 (48.00–53.00)	90.00 (89.00–90.50)	88.00 (88.00–90.00)	$p < 0.001$	$p < 0.001$	$p = 0.115$
	Q4	57.00 (55.50–61.50)	95.00 (92.00–97.50)	97.00 (93.50–99.00)	$p < 0.001$	$p < 0.001$	$p = 0.797$
**Total Progressive Motile Sperm Count (mill) Median (IQR) *n* = 100**	Q1	25.03 (15.98–28.71)	4.46 (3.09–5.69)	2.28 (1.57–2.77)	$p < 0.001$	$p < 0.001$	$p = 0.007$
	Q2	48.00 (35.45–56.53)	7.67 (6.74–8.85)	4.51 (3.99–5.22)	$p < 0.001$	$p < 0.001$	$p < 0.001$
	Q3	76.00 (66.64)	13.19 (11.71–14.64)	7.73 (6.78–8.69)	$p < 0.001$	$p < 0.001$	$p < 0.001$
	Q4	134.79 (101.98–149.92)	21.57 (19.39–25.12)	14.34 (11.34–18.79)	$p < 0.001$	$p < 0.001$	$p = 0.004$
**DNA Fragmentation Index (%) Median (IQR) DGC *n* = 90**	Q1	3.47 (3.08–3.92)	1.12 (0.52–1.47)	0.85 (0.41–1.34)	$p < 0.001$	$p < 0.001$	$p = 1.00$
	Q2	5.49 (5.13–6.27)	2.38 (2.03–2.80)	2.77 (2.16–3.63)	$p < 0.001$	$p < 0.001$	$p = 0.390$
	Q3	8.22 (7.25–9.40)	3.71 (3.45–4.35)	5.88 (5.08–6.83)	$p < 0.001$	$p = 0.002$	$p = 0.001$
	Q4	17.20 (14.20–20.48)	9.31 (6.17–11.75)	10.87 (9.39–15.02)	$p < 0.001$	$p = 0.004$	$p = 0.111$

* Quartile stratification of sperm quality parameters to compare SwimCount™ Harvester with Swim-up. a Statistically significant differences between unprocessed samples and samples processed by SwimCount™ Harvester. b Statistically significant differences between unprocessed samples and samples processed by Swim-up. c Statistically significant difference between SwimCount™ Harvester and Swim-up.

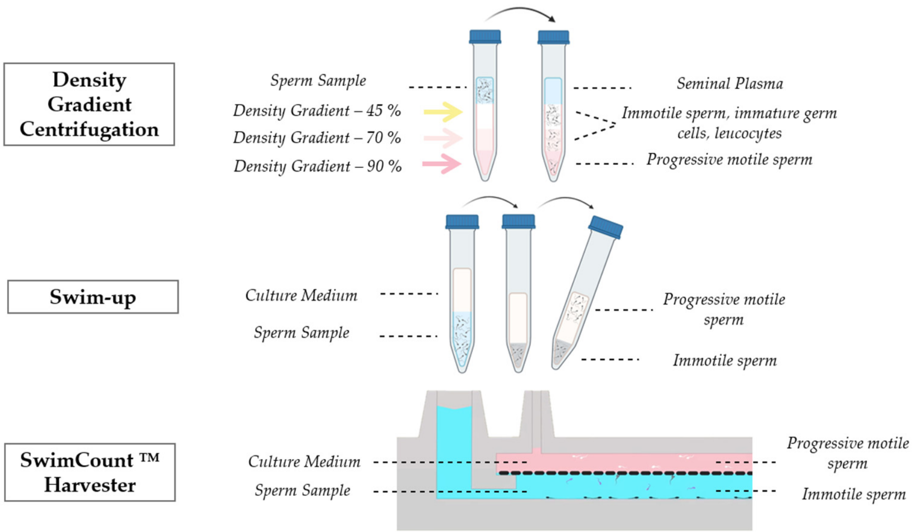

Figure S1. Graphical explanation of each sperm selection technique used in this study. Created with BioRender.com.
